# Supplementary figures and images for: Functional characterization of a ‘plant-like’ HYL1 homolog in the cnidarian Nematostella vectensis indicates a conserved involvement in microRNA biogenesis
Source: eLife. 2022 Mar 15;11:e69464. doi: 10.7554/eLife.69464 (PMC9098223; doi:10.7554/eLife.69464)

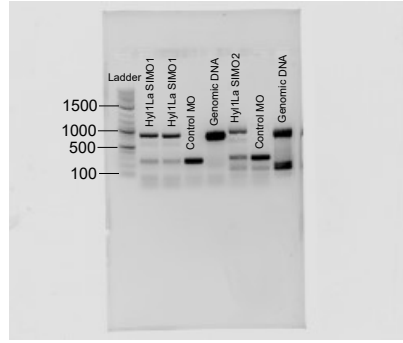

Supplement: Figure 2—figure supplement 1—source data 1. [file elife-69464-fig2-figsupp1-data1.zip › F2Sd1.pdf]

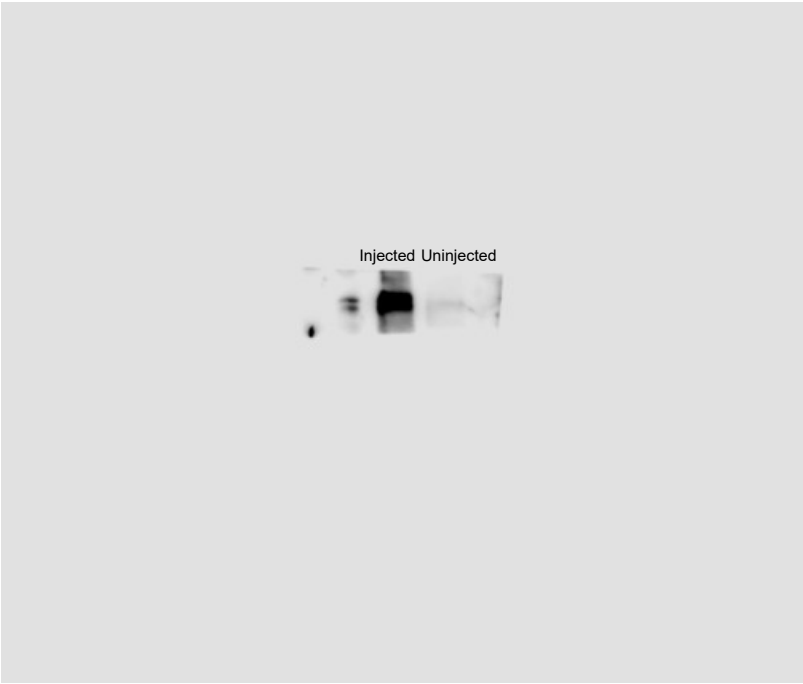

Supplement: Figure 5—source data 1. [file elife-69464-fig5-data1.zip › F5Sd1.pdf]

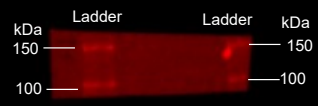

Supplement: Figure 5—source data 2. [file elife-69464-fig5-data2.zip › F5Sd2.pdf]

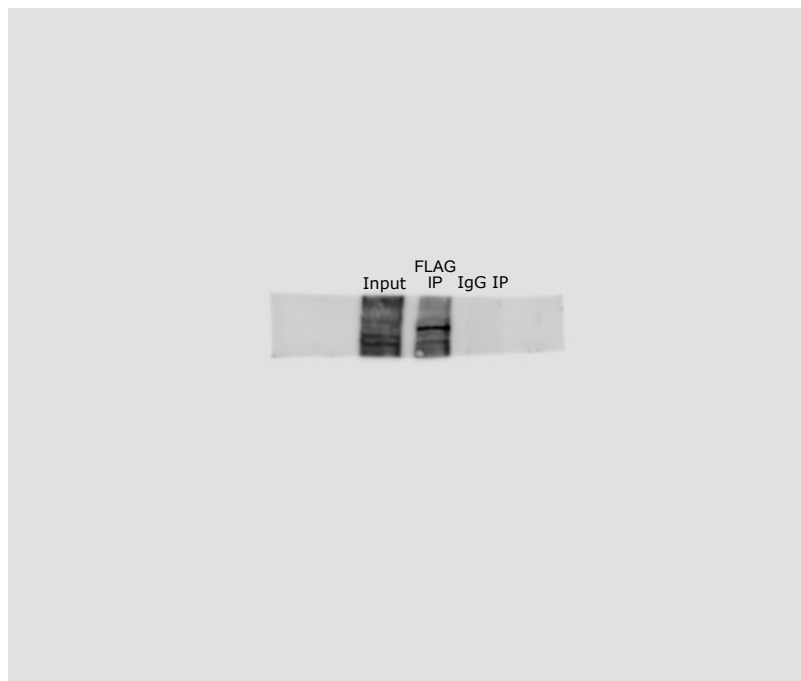

Supplement: Figure 5—figure supplement 1—source data 3. [file elife-69464-fig5-figsupp1-data3.zip › Fig5Sd3.pdf]

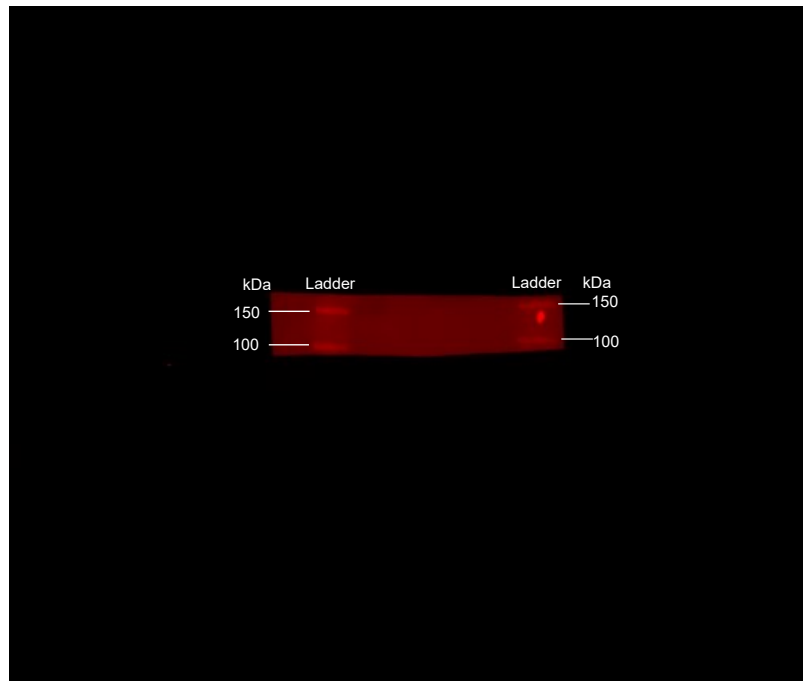

Supplement: Figure 5—figure supplement 1—source data 4. [file elife-69464-fig5-figsupp1-data4.zip › Fig5Sd4.pdf]

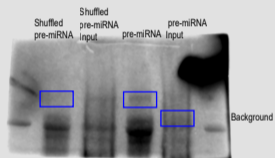

Supplement: Figure 6—source data 1. [file elife-69464-fig6-data1.zip › Fig6Sd1.pdf]

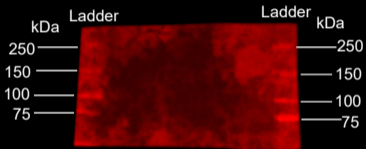

Supplement: Figure 6—source data 2. [file elife-69464-fig6-data2.zip › Fig6Sd2.pdf]

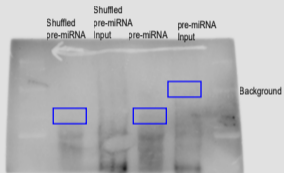

Supplement: Figure 6—source data 3. [file elife-69464-fig6-data3.zip › Fig6Sd3.pdf]

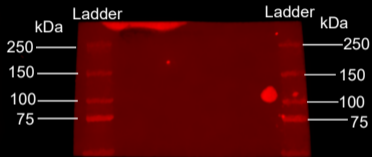

Supplement: Figure 6—source data 4. [file elife-69464-fig6-data4.zip › Fig6Sd4.pdf]

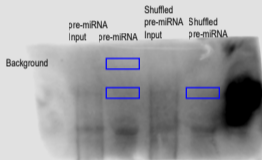

Supplement: Figure 6—source data 5. [file elife-69464-fig6-data5.zip › Fig6Sd5.pdf]

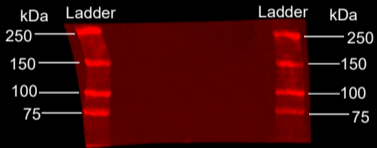

Supplement: Figure 6—source data 6. [file elife-69464-fig6-data6.zip › Fig6Sd6.pdf]

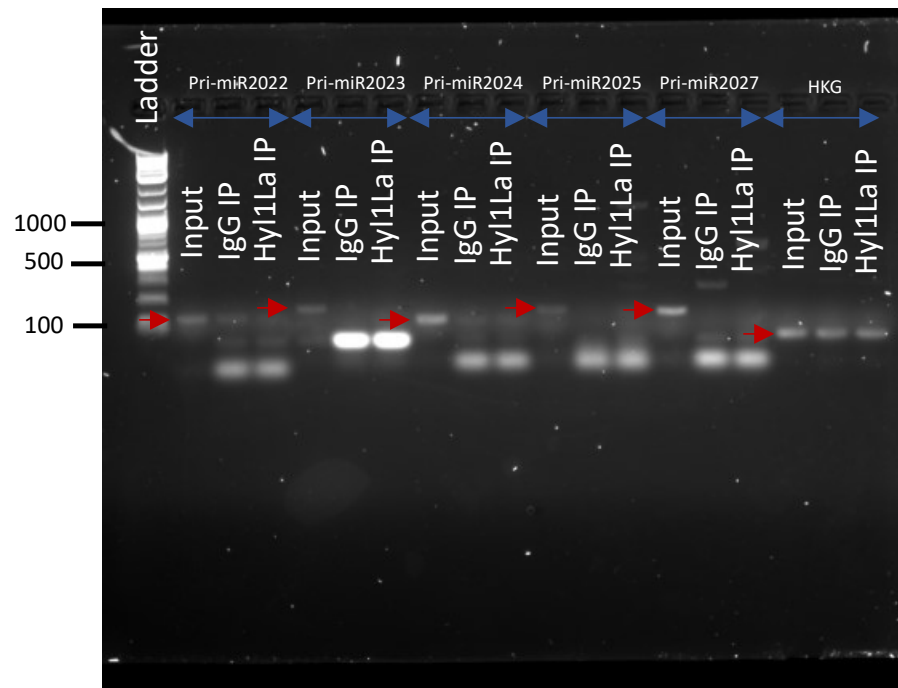

Supplement: Source data 1. [file elife-69464-data1.zip › Source_data files with labels/Figure_5_Source _data_5.pdf]

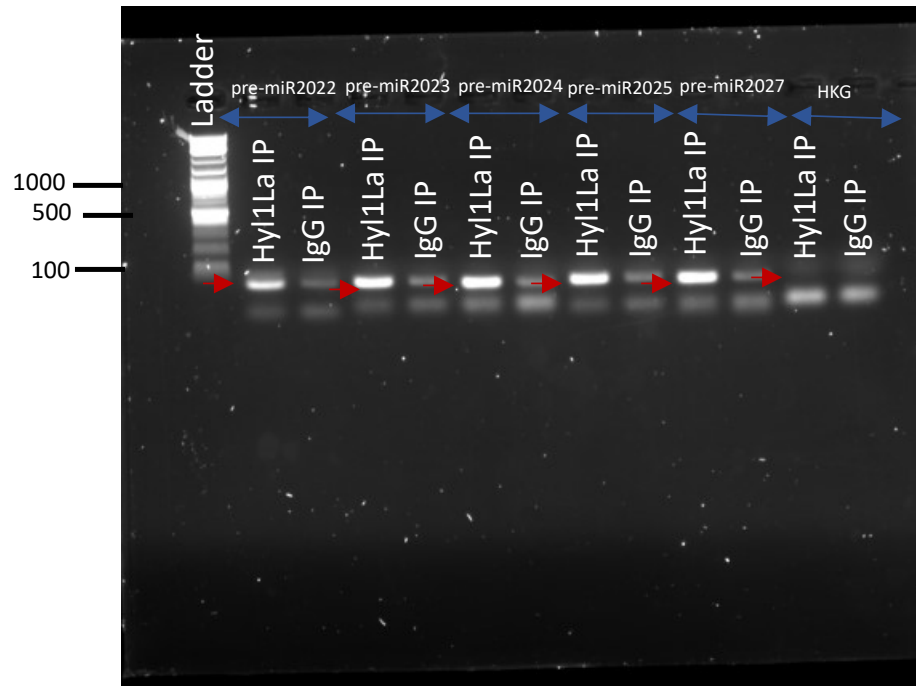

Supplement: Source data 1. [file elife-69464-data1.zip › Source_data files with labels/Figure_5_Source _data_6.pdf]

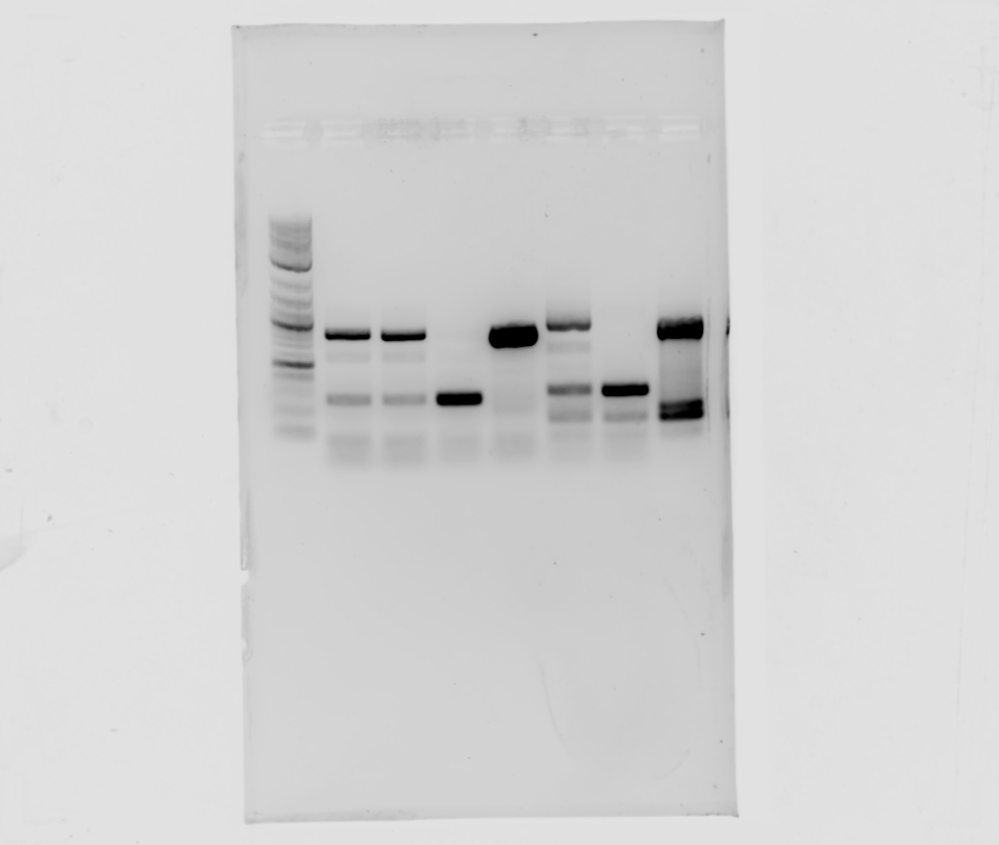

Supplement: Source data 2. [file elife-69464-data2.zip › Source data files without labels/Figure_2_Source _data_1.tif]

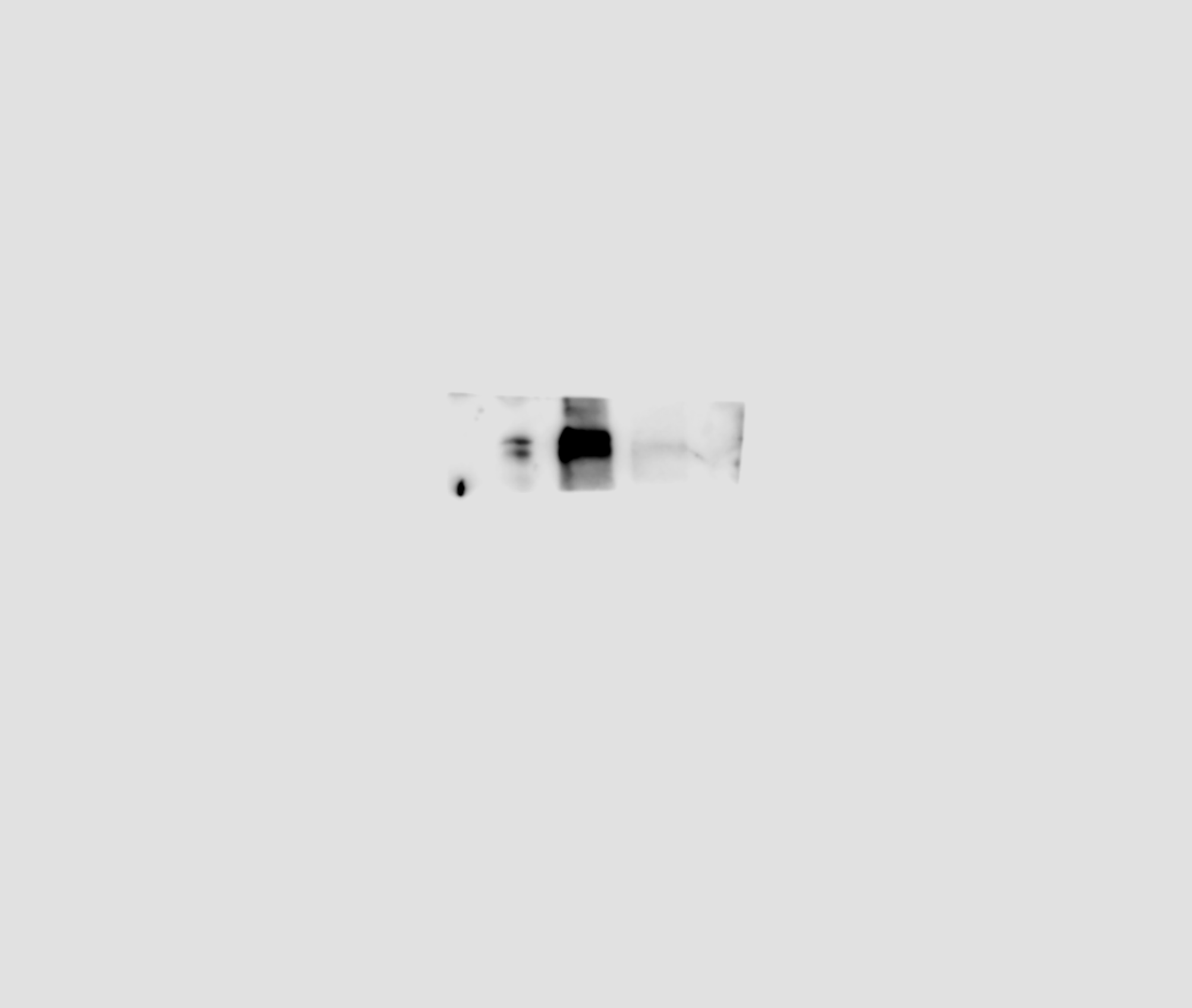

Supplement: Source data 2. [file elife-69464-data2.zip › Source data files without labels/Figure_5_Source _data_1.tif]

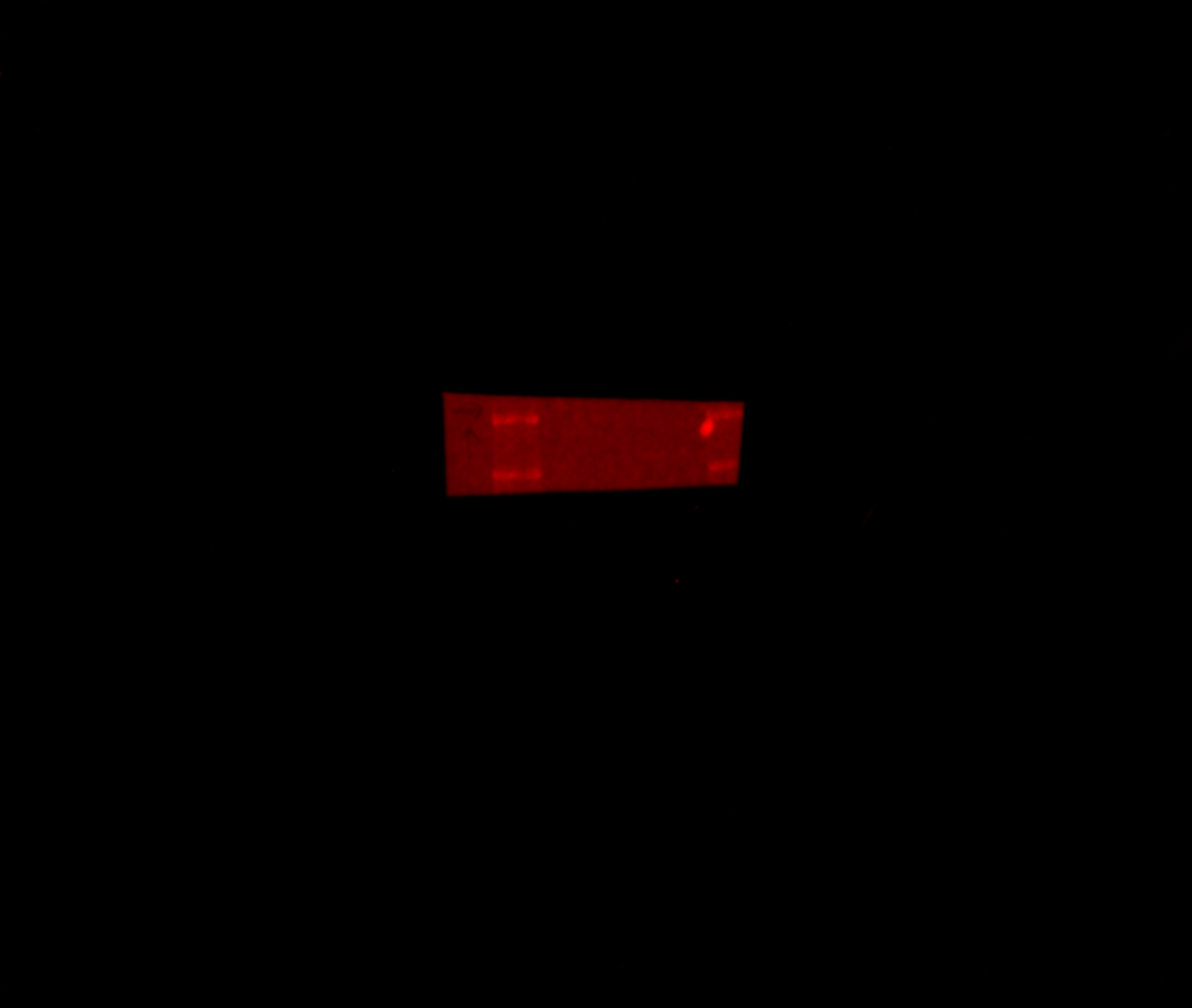

Supplement: Source data 2. [file elife-69464-data2.zip › Source data files without labels/Figure_5_Source _data_2.tif]

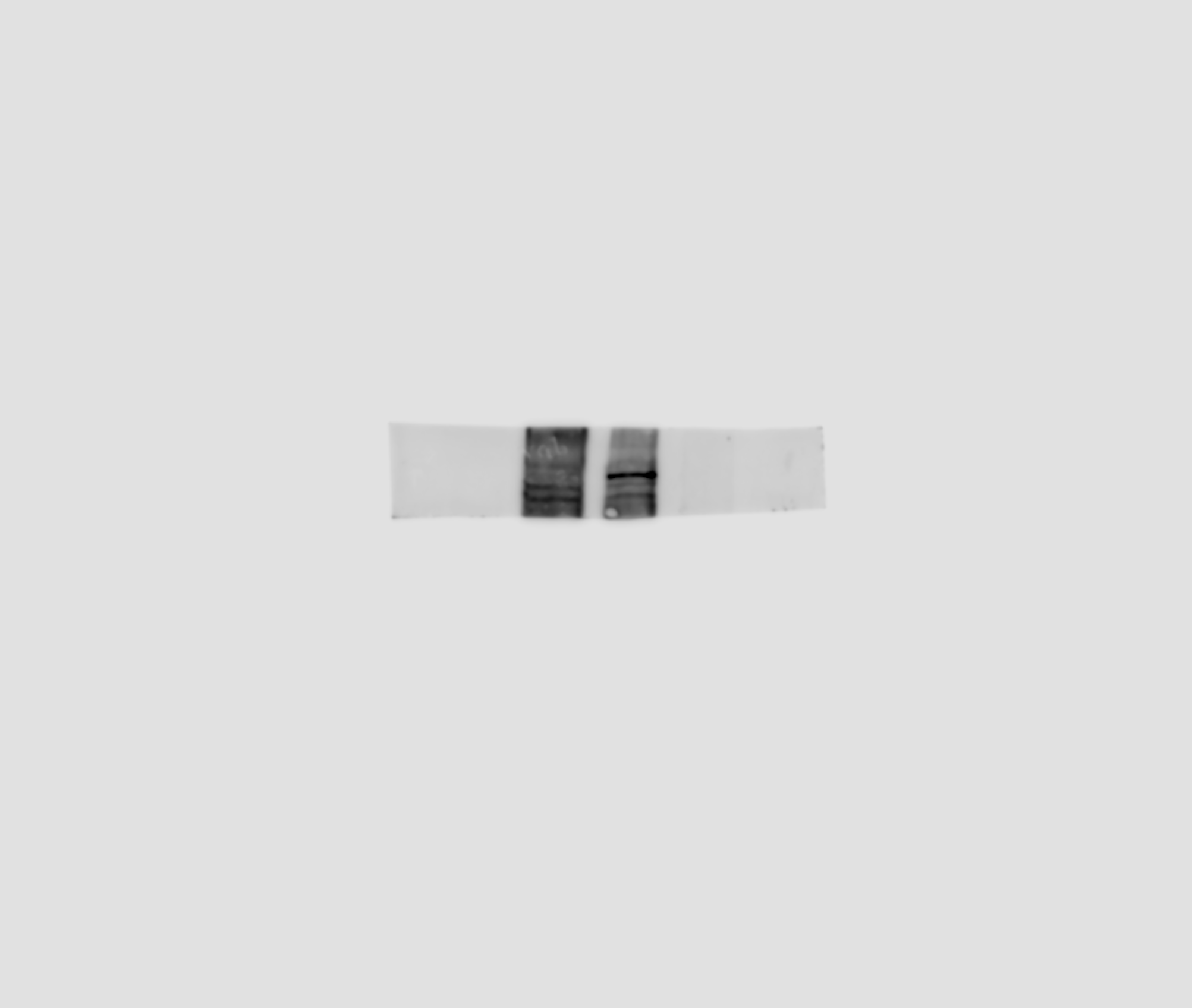

Supplement: Source data 2. [file elife-69464-data2.zip › Source data files without labels/Figure_5_Source _data_3.tif]

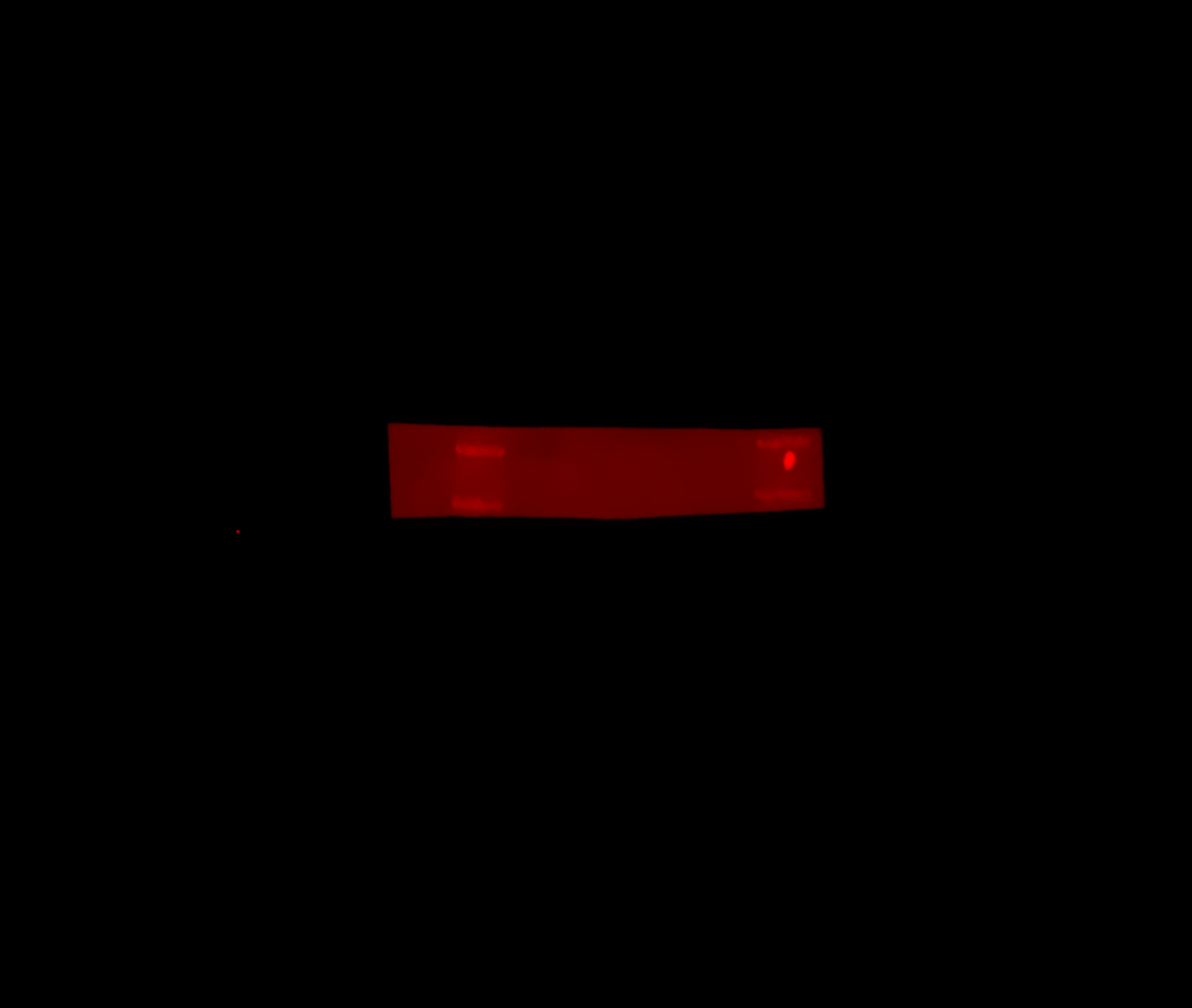

Supplement: Source data 2. [file elife-69464-data2.zip › Source data files without labels/Figure_5_Source _data_4.tif]

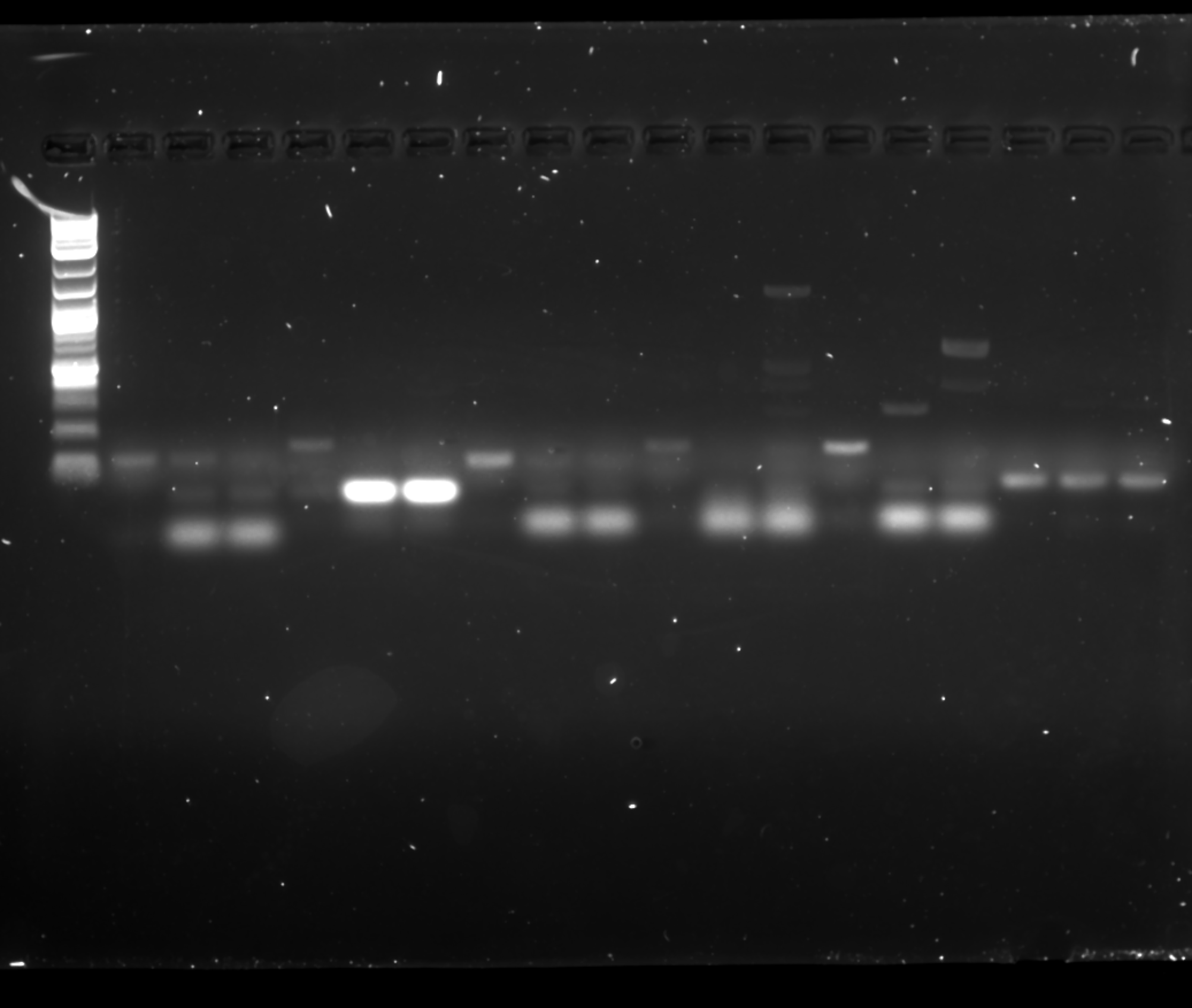

Supplement: Source data 2. [file elife-69464-data2.zip › Source data files without labels/Figure_5_Source _data_5.tif]

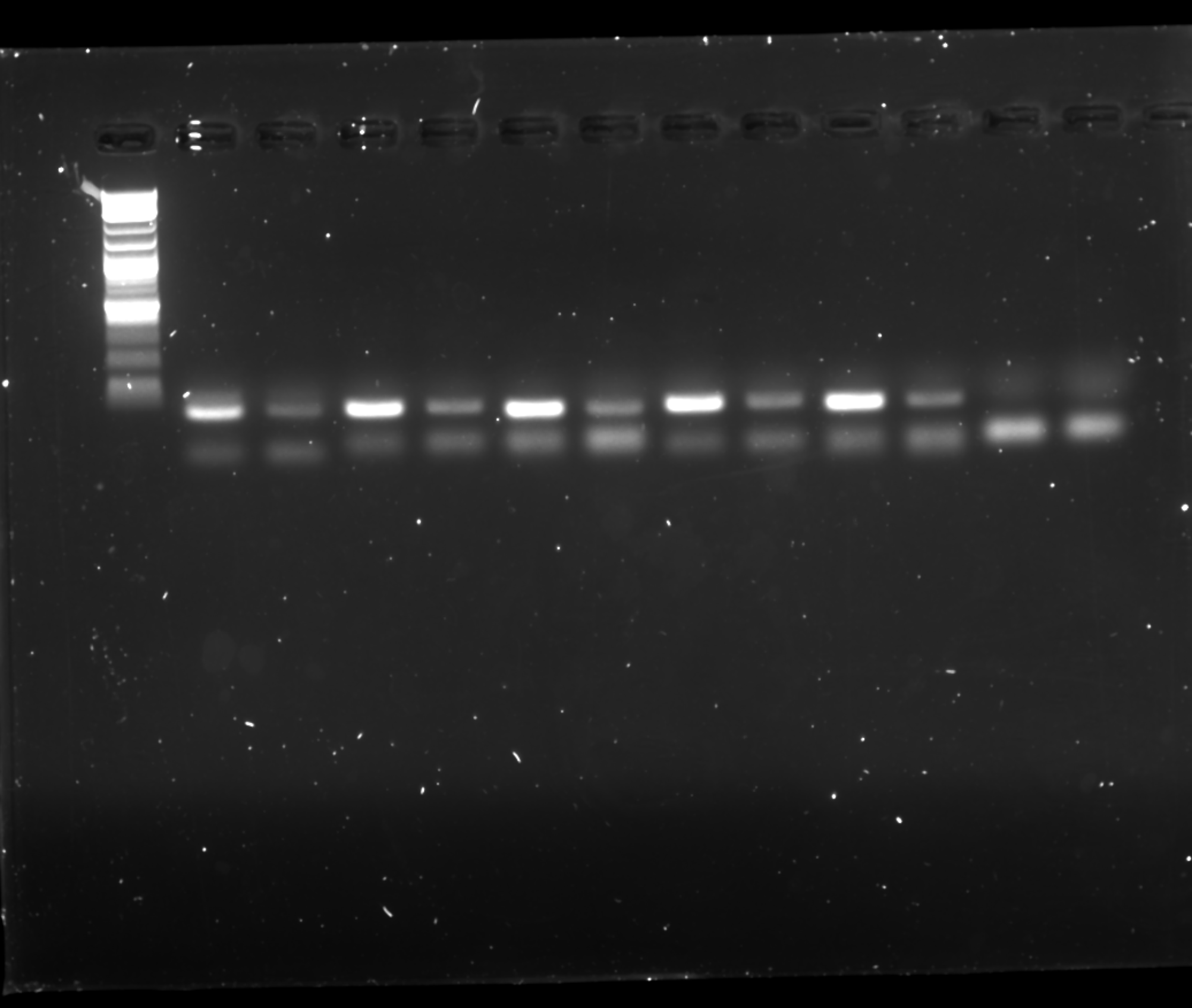

Supplement: Source data 2. [file elife-69464-data2.zip › Source data files without labels/Figure_5_Source _data_6.tif]

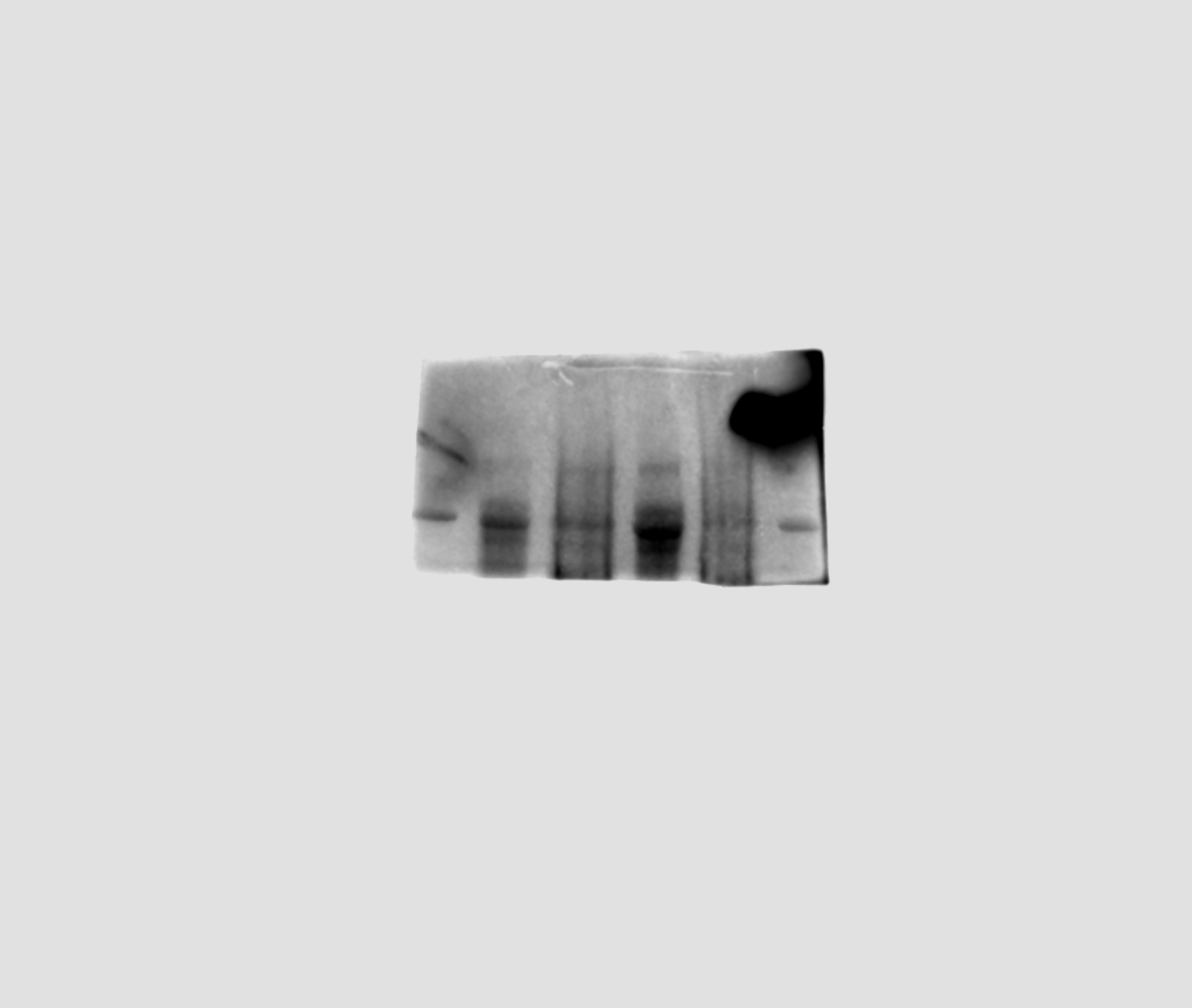

Supplement: Source data 2. [file elife-69464-data2.zip › Source data files without labels/Figure_6_Source _data_1.tif]

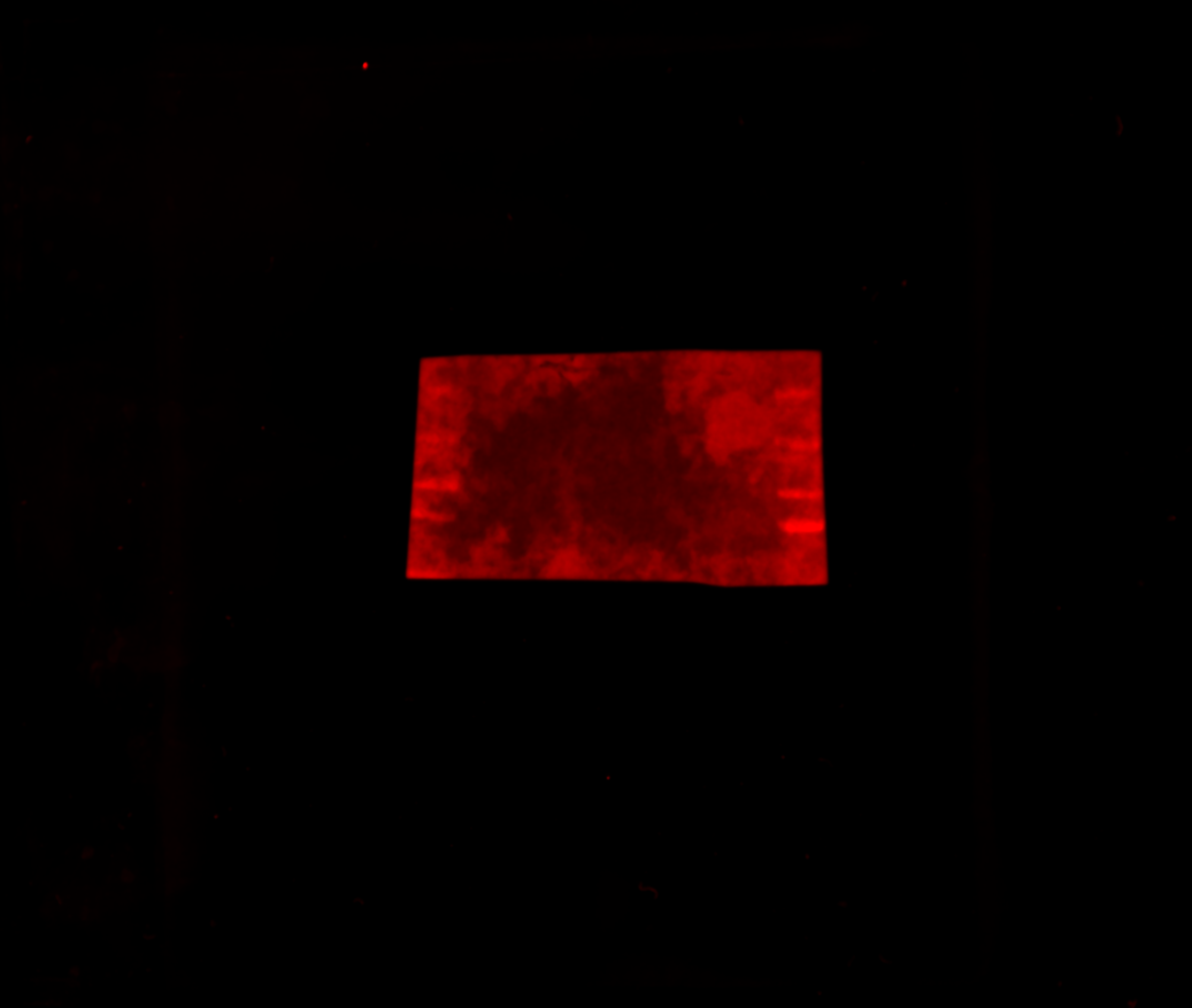

Supplement: Source data 2. [file elife-69464-data2.zip › Source data files without labels/Figure_6_Source _data_2.tif]

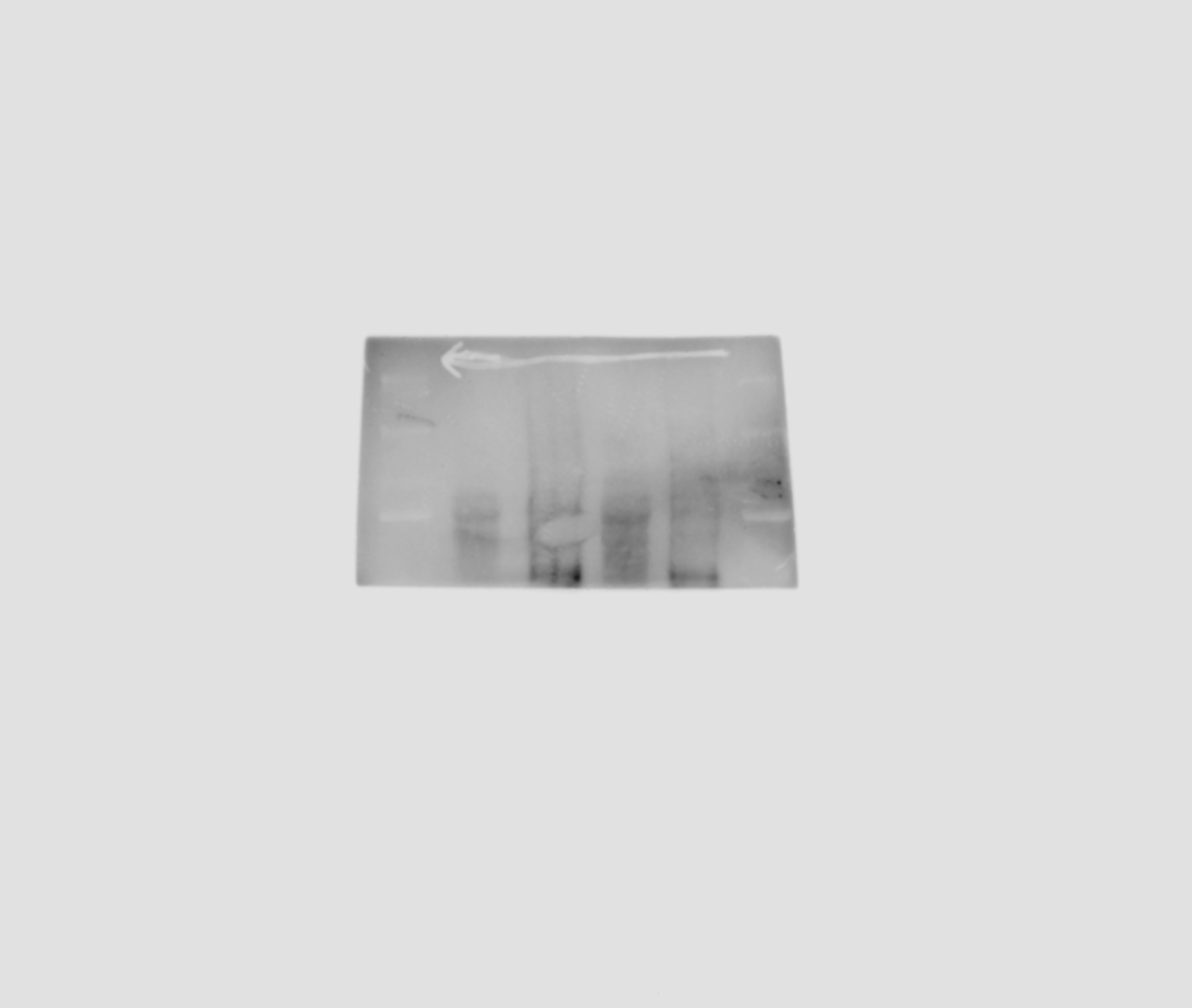

Supplement: Source data 2. [file elife-69464-data2.zip › Source data files without labels/Figure_6_Source _data_3.tif]

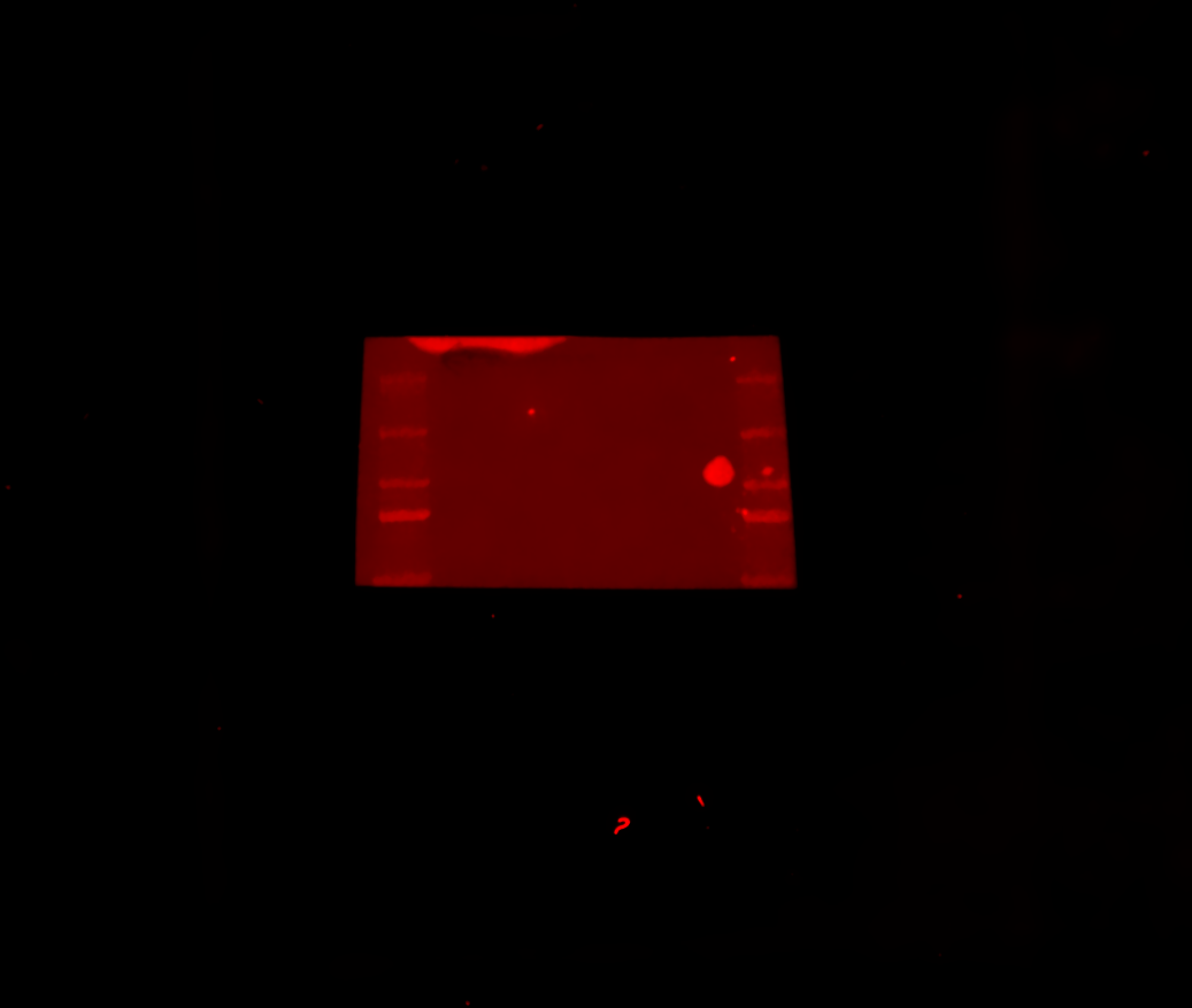

Supplement: Source data 2. [file elife-69464-data2.zip › Source data files without labels/Figure_6_Source _data_4.tif]

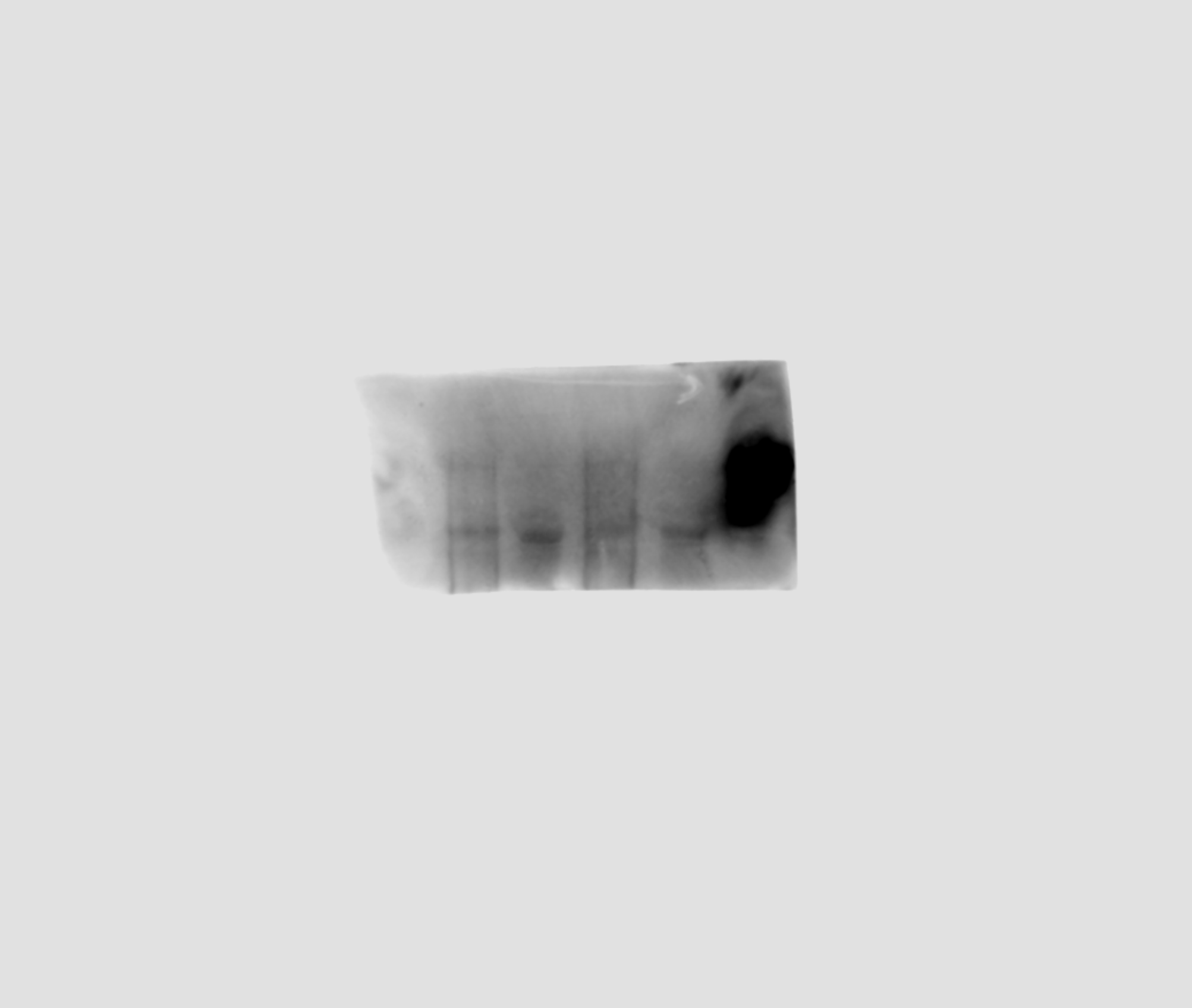

Supplement: Source data 2. [file elife-69464-data2.zip › Source data files without labels/Figure_6_Source _data_5.tif]

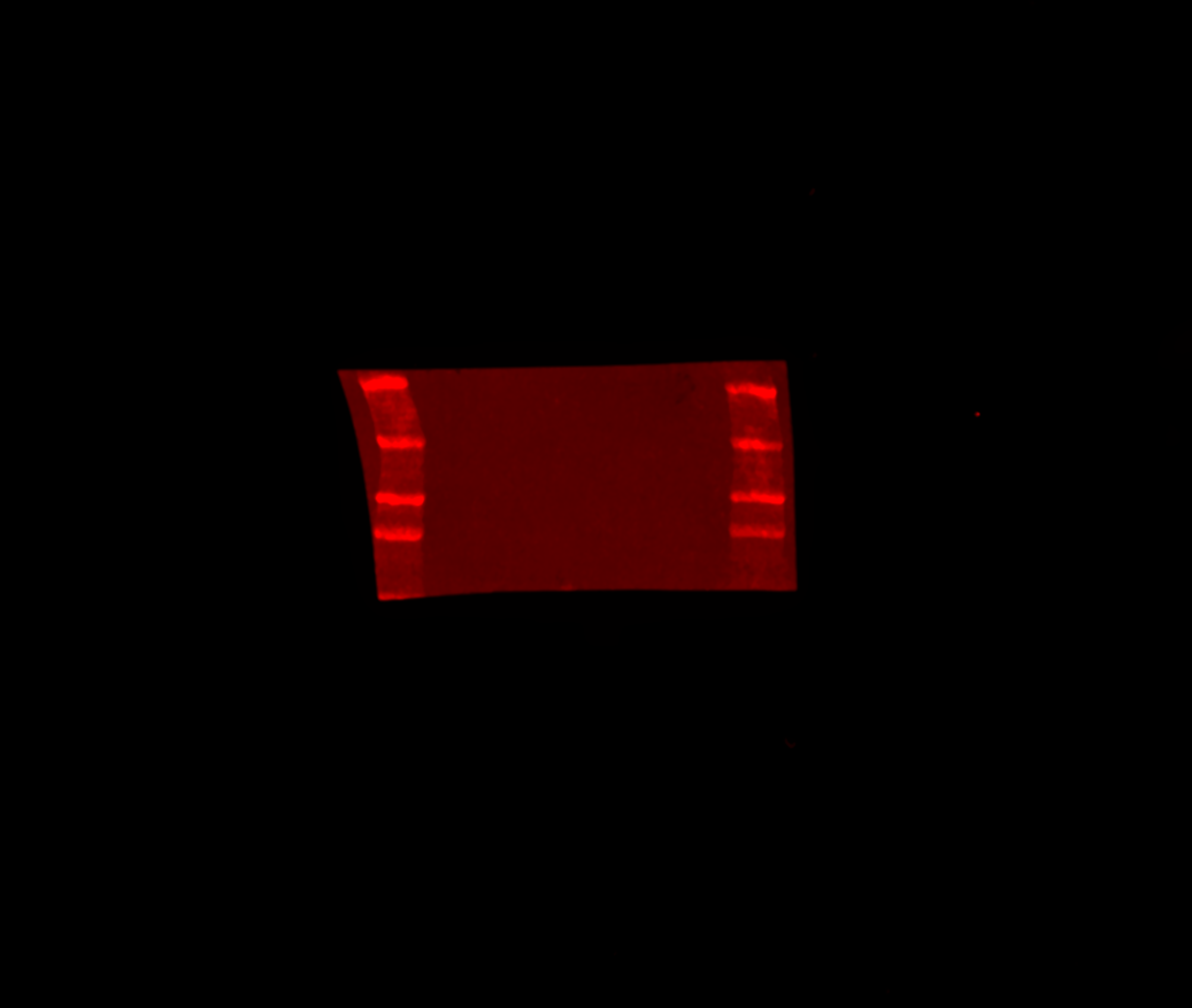

Supplement: Source data 2. [file elife-69464-data2.zip › Source data files without labels/Figure_6_Source _data_6.tif]
