# Supplementary material for: Functional characterization of a ‘plant-like’ HYL1 homolog in the cnidarian Nematostella vectensis indicates a conserved involvement in microRNA biogenesis
Source: eLife. 2022 Mar 15;11:e69464. doi: 10.7554/eLife.69464 (PMC9098223; doi:10.7554/eLife.69464)
Supplement: Transparent reporting form [file elife-69464-transrepform1.docx]

***eLife’s* transparent reporting form**

We encourage authors to provide detailed information *within their submission* to facilitate the interpretation and replication of experiments. Authors can upload supporting documentation to indicate the use of appropriate reporting guidelines for health-related research (see [EQUATOR Network](about:blank)), life science research (see the BioSharing Information Resource), or the [ARRIVE guidelines](about:blank) for reporting work involving animal research. Where applicable, authors should refer to any relevant reporting standards documents in this form.

If you have any questions, please consult our Journal Policies and/or contact us: [editorial@elifesciences.org](about:blank).

**Sample-size estimation**

- You should state whether an appropriate sample size was computed when the study was being designed
- You should state the statistical method of sample size computation and any required assumptions
- If no explicit power analysis was used, you should describe how you decided what sample (replicate) size (number) to use

Please outline where this information can be found within the submission (e.g., sections or figure legends), or explain why this information doesn’t apply to your submission:

In order to achieve statistical significance and to make the experiments manageable in a reasonable period of time, a sufficient number of technical and biological replicates were chosen.

Details of samples size (number of injected and uninjected animals) are mentioned in the “Materials and methods” section (line# 339-340, 460-461, 507-508). For phenotypic analysis the number of animals used are stated in Figure legend 2F.

No explicit power analysis was performed as it was irrelevant for the study.

**Replicates**

- You should report how often each experiment was performed
- You should include a definition of biological versus technical replication
- The data obtained should be provided and sufficient information should be provided to indicate the number of independent biological and/or technical replicates
- If you encountered any outliers, you should describe how these were handled
- Criteria for exclusion/inclusion of data should be clearly stated
- High-throughput sequence data should be uploaded before submission, with a private link for reviewers provided (these are available from both GEO and ArrayExpress)

Please outline where this information can be found within the submission (e.g., sections or figure legends), or explain why this information doesn’t apply to your submission:

The details of replicates used in this study are available in the “Results" and Materials and methods” sections (line# 207, 228, 347-350, 372-373, 431-432), in the figure legends of Figure 3B-D, Figure 3 - figure supplement 1, Figure 3 - figure supplement 2 (A-D), Figure 4D, Figure 5D, Figure 5A, Figure 6D and in Supplementary file 2, Supplementary file 3, Supplementary file 4, Supplementary file 5, Supplementary file 6.

All the experiment were performed with three biological as well as two technical replicates for the qPCR.

The distinct batches of animals are defined as biological replicates while distinct samples from same batch are defined as technical replicates (line# 347-349).

We did not encounter any outliers.

The small RNA and total RNA sequencing data are submitted to NCBI-SRA under BioProject ID PRJNA630340 and is freely available at [https://www.ncbi.nlm.nih.gov/bioproject/PRJNA630340](about:blank). Line# 584

**Statistical reporting**

- Statistical analysis methods should be described and justified
- Raw data should be presented in figures whenever informative to do so (typically when N per group is less than 10)
- For each experiment, you should identify the statistical tests used, exact values of N, definitions of center, methods of multiple test correction, and dispersion and precision measures (e.g., mean, median, SD, SEM, confidence intervals; and, for the major substantive results, a measure of effect size (e.g., Pearson's r, Cohen's d)
- Report exact p-values wherever possible alongside the summary statistics and 95% confidence intervals. These should be reported for all key questions and not only when the p-value is less than 0.05.

The details of statistical analysis can be found in the “Materials and methods” section (line# 533-541). We have used Student's t-test, and Wilcoxson’s rank test in the study. We ensure that we have presented raw data in the figures where ever possible. The p values, N and SD for each statistical analysis is given in the “Results” section as well as in the figures legends. The figure (main or supplementary) representing a mean value is always accompanied by bar representing SD. No multiple correction was required.

Please outline where this information can be found within the submission (e.g., sections or figure legends), or explain why this information doesn’t apply to your submission:

(For large datasets, or papers with a very large number of statistical tests, you may upload a single table file with tests, Ns, etc., with reference to sections in the manuscript.)

**Group allocation**

- Indicate how samples were allocated into experimental groups (in the case of clinical studies, please specify allocation to treatment method); if randomization was used, please also state if restricted randomization was applied
- Indicate if masking was used during group allocation, data collection and/or data analysis

Please outline where this information can be found within the submission (e.g., sections or figure legends), or explain why this information doesn’t apply to your submission:

The animals injected with different morpholinos and shRNA that interact with the target gene (Hyl1La) were considered as treatment group while animals injected with control morpholino and shRNA (no acting site in *Nematostella* genome) were considered as control group. This has been clearly defined in the “Results” section (line# 115-116, 186-190).

As part of the IP experiment, samples precipitated with 3xFLAG-Hyl1La are considered as treatment, while samples precipitated with whole mouse IgG are considered as control (Figure 5 legend).

For in vitro binding assays, samples pulled down with miR-2022 backbone are considered treatment groups, while samples pulled down with shuffled miR-2022 backbones are considered control groups. This has been clearly defined in the “Materials and methods” section line# 492-495.

**Additional data files (“source data”)**

- We encourage you to upload relevant additional data files, such as numerical data that are represented as a graph in a figure, or as a summary table
- Where provided, these should be in the most useful format, and they can be uploaded as “Source data” files linked to a main figure or table
- Include model definition files including the full list of parameters used
- Include code used for data analysis (e.g., R, MatLab)
- Avoid stating that data files are “available upon request”

Please indicate the figures or tables for which source data files have been provided:

Source data files have been provided for Figure 2, Figure 5, and Figure 6.
